# Case Report: A diagnostic dilemma: metachronous pancreatic metastasis from high-grade serous ovarian carcinoma

**DOI:** 10.3389/fonc.2026.1901559

**Published:** 2026-08-12

**Authors:** Bahaaeldin Baraka, Maryam Al-Ani, Adam Afife, Irshad Soomro, Srinivasan Madhusudan

**Affiliations:** 1 Department of Medical Oncology, Division of Cancer, City Hospital, Nottingham University Hospitals, Nottingham, United Kingdom; 2 Health Education Yorkshire and Humber, NHS England, Leeds, United Kingdom; 3 Department of Histopathology, City Hospital, Nottingham University Hospitals, Nottingham, United Kingdom; 4 Naaz-Coker Ovarian Cancer Research Centre, Biodiscovery Institute, School of Medicine, University of Nottingham, Nottingham, United Kingdom

**Keywords:** advanced ovarian cancer, case report, chemotherapy, cytoreductive surgery, high-grade serous carcinoma, metachronous metastasis, oncology, ovarian cancer

## Abstract

**Background:**

Ovarian cancer, a leading cause of gynecological cancer mortality, rarely metastasizes to the pancreas. This review seeks to consolidate knowledge about metachronous pancreatic metastases from ovarian cancer, emphasizing clinical presentations, diagnostic challenges, treatment options and outcomes.

**Methods:**

Following PRISMA guidelines, a systematic literature search was conducted in PubMed, EMBASE, and the Cochrane library between 1989 and December 2023. Only studies with histological proof of ovarian cancer metastasis to the pancreas were considered.

**Results:**

We identified 7 studies that reported a total of 8 cases. Most metastases originated from a primary high-grade serous carcinoma. The treatment options varied and included surgical excision and chemotherapy; we considered both radiological and histological outcomes.

**Conclusions:**

Although rare, metachronous pancreatic metastases from ovarian cancer pose a unique clinical challenge. Elucidating this possible metastatic pathway may enhance patient outcomes and help identify the most appropriate management.

## Introduction

Ovarian cancer is a significant health concern in the UK, with approximately 7,500 new cases annually (2016-2018) (1). In women, it accounts for 4% of the total number of newly diagnosed cancer cases, making it the sixth most frequent malignancy ^1^. Since the early 1990s, the incidence rate has remained consistent but there has been a positive trend in the last decade with a 5% decrease. Over 25% of cases each year occur in women aged 75 and over. Projections indicate a 5% increase in incidence rates by 2038-2040, reaching around 9,400 new cases annually (1). Despite advances in imaging and CA125 measurement for cancer detection, over 70% of newly diagnosed epithelial ovarian cancer (EOC) patients present with advanced disease beyond the pelvic area (FIGO stages III or IV) due to nonspecific symptoms. Prior to 2006, approximately one-third of EOC cases in England were emergency presentations, up to 74% of these patients did not receive any active cancer treatment^2^. Demographically, incidence rates in England showed no significant variation by deprivation quintile, but lower rates are observed in certain ethnic groups compared to the white ethnic group (2013-2017). Despite these challenges, survivorship is evident, with an estimated 41,000 women previously diagnosed with ovarian cancer were still alive in the UK by the end of 2010. These data highlight the ongoing need for research, awareness and targeted interventions in the battle against ovarian cancer (2). Ovarian cancer most commonly disseminates within the peritoneal cavity rather than through early hematogenous spread. Tumor cells may exfoliate from the ovarian surface, survive within ascitic fluid, and implant on peritoneal surfaces, particularly the omentum, diaphragm, bowel serosa, and pelvic peritoneum. Lymphatic spread, including retroperitoneal nodal involvement, is also recognized, while distant visceral metastases are less frequent and usually reflect advanced disease. (3, 4).

Among its metastatic presentations, pancreatic involvement is exceptionally rare, posing diagnostic and therapeutic challenges. When it occurs, the route of spread may be difficult to determine with certainty and may include transcoelomic, lymphatic, or hematogenous mechanisms. Here, we present a case of metastasis to the pancreas from high grade papillary serous ovarian cancer and a literature review.

## Case presentation

A 69-year-old woman presented in January 2017 with vague symptoms of abdominal bloating, discomfort, and ascites. Initial laboratory work-up showed a markedly elevated CA125 level of 2759 U/mL. She underwent diagnostic laparoscopy, omental biopsy and ascitic drainage in Feb 2017. Histology confirmed high-grade papillary serous adenocarcinoma of ovarian origin, FIGO stage IIIC. Germline BRCA testing was negative.

At diagnosis, she was otherwise fit and active, with no major comorbidities and a WHO performance status of 1. Her only regular medication was amitriptyline, which had been prescribed for sleep disturbance following her diagnosis.

Subsequent to the gynaecological multidisciplinary team discussion, she was planned to have three cycles of neoadjuvant chemotherapy with carboplatin, paclitaxel, and bevacizumab, followed by re-imaging and consideration of subsequent cytoreductive surgery. The three pre-operative chemotherapy cycles were completed uneventfully after which she underwent successful optimal debulking surgery, followed by a further three cycles of the same chemotherapy regimen in the adjuvant setting and maintenance bevacizumab therapy.

In May 2018, she completed bevacizumab maintenance with fair tolerability and insignificant adverse events. Follow-up imaging in July 2019 showed no evidence of recurrent or metastatic disease and CA125 remained within normal value of 30 U/mL. However, in December 2020, CT imaging demonstrated a small tumor deposit anterior to the rectum indicating small volume disease recurrence. Given the long platinum-free interval of more than 12 months, she was re-treated with carboplatin and paclitaxel. A CT scan in April 2021 showed a good partial response. Following review by the gynaecological team, further surgery was not recommended and systemic treatment was continued. By July 2021, she had completed six cycles of carboplatin and paclitaxel, achieving a complete radiological response. She commenced maintenance rucaparib in August 2021.

**Figure 1:**
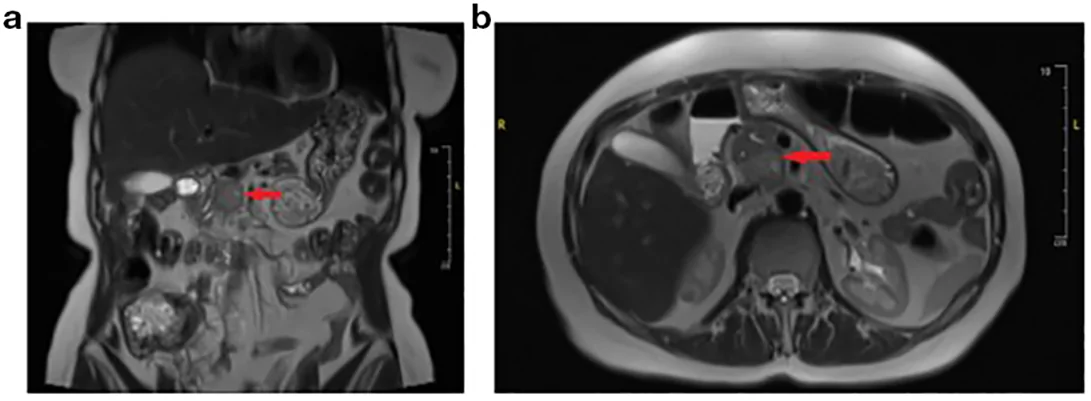
**(a)** T2 Abdomen MRI, coronal section. Pancreatic metastatic lesion of ovarian cancer (red headed arrow). **(b)** T2 Abdomen MRI, axial section. Pancreatic metastasizes (red headed arrow).

In January 2023, her CA125 level exhibited an upward trend. A non-contrast CT scan of the chest, abdomen, and pelvis (down to her known allergy to iodine contrast) did not show radiological evidence of disease progression. Following multidisciplinary discussion, treatment with rucaparib was continued. In March 2023, MRI was performed due to the uptrend in CA125 despite the absence of clear disease progression on CT, however it showed a soft tissue mass in the precaval region (**Figure 1**). Her case was subsequently reviewed by the hepatopancreatobiliary (HPB) multidisciplinary team; the main differential diagnoses were focal recurrence versus a primary tumor arising from the uncinate process of the pancreas.

**Figure 2:**
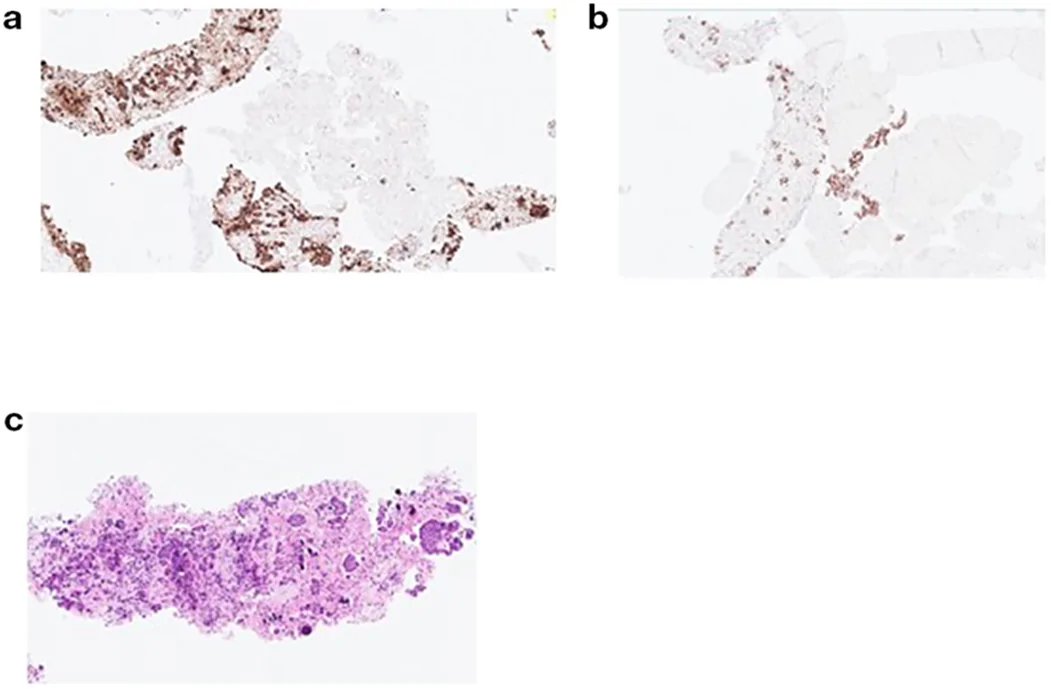
**(a)** CK7 positive, 100x magnification. **(b)** WT1 positive, 100x magnification. **(c)**. High grade papillary serous carcinoma with papillary structures and psammoma bodies. H&E stain 200x magnification.

On 28 April 2023, she underwent an endoscopic ultrasound-guided biopsy of the pancreatic lesion. Histopathological analysis confirmed metastatic serous papillary carcinoma of ovarian origin (**Figure 2**).

In June 2023, she underwent an endoscopic retrograde cholangiopancreatography (ERCP) for a 20 mm common bile duct stricture. The procedure entailed a pancreatic duct stent placement.

Between June and October 2023, she received six cycles of carboplatin and liposomal doxorubicin with palliative intent. CT scan in December 2023 showed stable disease. Biochemically, CA125 levels were stable. Nevertheless, in February 2024, CA125 demonstrated a resurgence suggesting biochemical progression. Weekly paclitaxel was started in April 2024. Intolerable treatment related toxicity concomitant with general health decline had precluded further active oncological treatment and subsequently the focus of management transitioned to best supportive care led by palliative care team.

During palliative care follow-up, she reported persistent fatigue, weight loss, reduced appetite and intermittent abdominal pain. Thereafter, she developed cholangitis, likely related to previous biliary intervention, with fever, rigors and elevated liver enzymes including ALT and ALP. Management was conservative with antibiotics and other supportive measures. Her wishes were against chemotherapy but with continuous supportive care. She gradually deteriorated, with increasing frailty and cachexia requiring optimization of analgesia and domiciliary care. She sadly passed away in July 2024 under the care of the community palliative care team. Overall survival from the initial diagnosis of ovarian cancer was approximately 90 months, while survival from the diagnosis of pancreatic metastasis was approximately 15 months.

This case highlights the complex progression of high-grade serous ovarian carcinoma, including an uncommon metastatic spread to the pancreas. Despite initial optimal debulking surgery and a favorable response to first-line chemotherapy, the patient’s course underscores the importance of a multidisciplinary approach, integrating surgical, oncological and palliative care services, especially in the advanced stages of ovarian cancer with atypical metastatic patterns.

## Materials and methods

This systematic review followed the PRISMA principles. A structured and thorough literature search was conducted across PubMed, Embase, and the Cochrane Library. The search covered studies published between 1989 and 2023 to capture all the reported data in this context. Search terms included combinations of keywords and controlled vocabulary such as metastatic,” “ovarian,” “pancreas,” and “cancer”. To refine our search outcome, we used boolean operators (and/or).

### Study eligibility

1. Studies were included if they involved histopathological confirmation of pancreatic metastasis from ovarian cancer, either symptomatic or asymptomatic.
2. All histological subtypes of ovarian cancer were considered, provided that ovarian malignancy was confirmed as the primary tumor site.
3. Eligible study designs included case reports, case series, retrospective cohort studies, prospective studies and randomized controlled trials.

### Exclusion criteria

1. Studies that failed to confirm nature of pancreatic lesion via histopathology examination.
2. Trial protocols.
3. Conference abstracts.
4. Articles that were not available in full text were excluded.
5. Studies published in languages other than English.

### Data collection

The following variables were extracted using a predefined data extraction file: publication details (i.e., study title, authors, publication date/year and study design), patient characteristics if available (i.e., number of patients, age, primary tumor stage (FIGO stage), site of pancreatic metastases and other sites of metastatic disease) and survival.

### Data synthesis and statistical analysis

Due to the small number of included cases, heterogeneity in study design and incomplete reporting of clinical outcomes, meta-analysis was not feasible. Therefore, a descriptive synthesis was performed.

Baseline characteristics were summarised at the individual study level. Categorical variables, such as study design, site of pancreatic involvement and presence of extra-pancreatic disease, were reported as frequencies where possible. Continuous variables, including patient age and tumor size, were summarised using ranges because individual patient-level data were limited. Survival outcomes were reported descriptively, based on the available follow-up data.

Given the diverse patient populations and the infrequent nature of the condition being studied, along with the variability in the types of data reported (such as exact sizes and survival times), the descriptive statistics were designed to offer a comprehensive summary rather than a detailed numerical analysis. This method recognizes the constraints presented by the various data formats and the limited sample sizes commonly found in case-focused studies.

## Results

**Figure 3:**
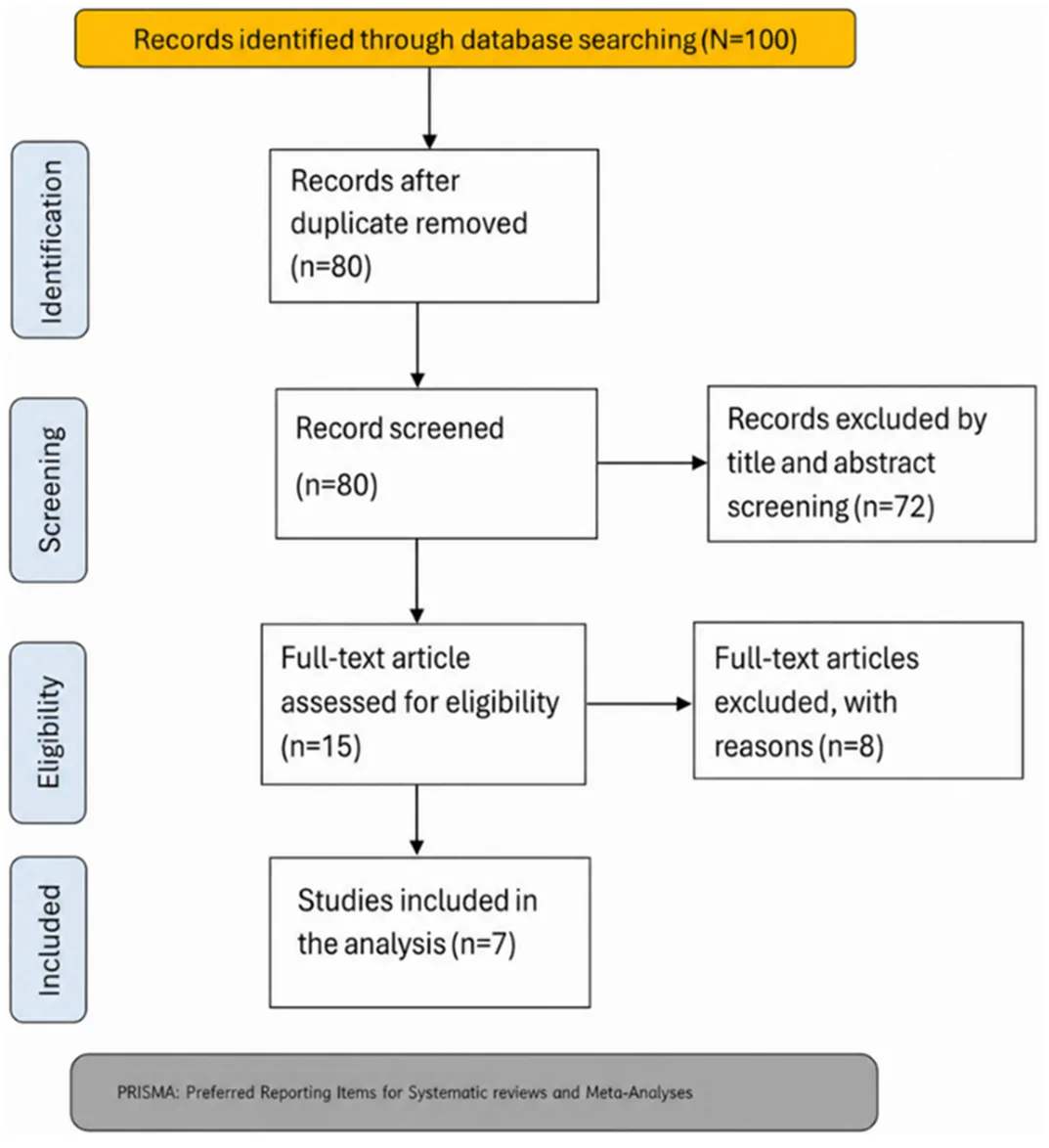
PRISMA flow diagram. PRISMA, preferred reporting items for systematic reviews and meta-analyses.

We identified a total of 15 studies through the literature search. Following screening titles and abstracts, 10 articles were selected for full-text review. Of these, one article was excluded because the full text was not available. Another was excluded because it was not published in English. Full-text assessment resulted in identification of seven studies that met the inclusion criteria and were included in this systematic review. The study selection process is summarised in the PRISMA flow diagram shown in **Figure 3**.

## Study characteristics

The seven included studies consisted of four case reports, two retrospective cohort studies, and one case series, as shown in **Table 1**. Across these studies, pancreatic lesions were confirmed histologically, supporting the diagnosis of metastatic disease rather than a primary pancreatic malignancy.

**Table 1:** Summary of published cases of pancreatic metastasis from ovarian malignancy included in the literature review.

Reference	Study design	Age	No. of cases	Previous history of malignancy	Site of pancreatic metastases	Extra-pancreatic metastasis	Tumor size	Endoscopic ultrasound/imaging characteristics	Immunohistochemistry	Survival/follow-up
(5)	Case report	30	1	Dysgerminoma (right salpingo-oophorectomy 14 years prior)	Head of Pancreas	Second part of the duodenum and liver	Nodules 1–2 cm, duodenal stricture	Thickening of the second part of the duodenum, dilated common bile duct	Infiltrating tumor with vesicular nucleus, prominent nucleoli, and distinct cell margins	Survived 5 years until 2001, no more follow up is available.
(6)	Case report	70	1	Bilateral hysteroannesiectomy for ovarian and uterine serous papillary adenocarcinoma G3 (pT1c N0)	Head of pancreas	None	2.5 cm × 3 cm	Hypodense lesion involving the portal vein, CBD stenosis	Adenocarcinoma with CA 125 expression	N/A
(7)	retrospective cohort study	61	1 out of 29 patients	Ovarian ca with Diaphragm, lymph nodes metastases	tail	Spleen, duodenum	N/A	N/A	N/A	42
(8)	A case series	53	1 out of five cases	High-grade serous ovarian carcinoma	Head and neck	N/A	3.9 × 3.8 cm	Hyperechoic heterogeneous with irregular borders Invasion to pancreatic duct and distal common bile duct	PAX8 positive, WT1 positive	N/A
(9)	A retrospective cohort study		2 out of 27 patients	N/A	N/A	N/A	N/A	N/A	N/A	The longest survival of the cohort was 26 months.
(10)	A case report	60	1	stage IIIC ovarian carcinoma.	peripancreatic region especially at pancreatic head region, splenic hilum and adjacent to the transverse colon area.	the head and body of the pancreas.	the largest measuring 4.7 x 3.6cm.	pseudocysts; CT scan showed multiple lesions in peripancreatic regions.	positive for CK7, negative for CK20, and CA 19–9 level at 101.5.	
(11)	A case report	51	1	Ovarian papillary serous cystadenocarcinoma (stage IV)	Body of Pancreas	Stomach	1.0×1.0-cm	A 4.6×4.2-cm intramural mass in the gastric antrum,and A 1.0×1.0-cm mass in the pancreatic body.	positivity for cytokeratin 7 (++), CA-125 (+), estrogen receptor (+ +), progesterone receptor (+), and CD56 (+ +), and negativity for cytokeratin 20 (–) and CDX-2 (–).	N/A

Overall, the included studies reported 67 cases of pancreatic metastases from different primary tumors. Among these, eight patients had pancreatic metastases confirmed to have originated from ovarian cancer. This small number highlights the rarity of pancreatic involvement in ovarian malignancy. The age ranged from 30 to 70 years for these 8 patients. The reported sites of pancreatic involvement varied across the cases and included the head, body, tail and peripancreatic region. In several cases, extra-pancreatic disease was also present, including metastases involving the liver, duodenum, spleen, stomach, lymph nodes and diaphragm. Tumor size was inconsistently reported, ranging from 1.0 × 1.0 cm to 4.7 × 3.6 cm in studies where this information was available.

Histological and immunohistochemical confirmation was central to diagnosis in the included reports. Reported biomarkers included the usual and fundamental makers such as CA-125 expression, CK7 positivity, WT1 positivity and PAX8 positivity, supporting an ovarian origin in the reported cases. However, detailed immunohistochemical data were not available in all studies.

Survival outcomes were variably reported (5), while Chikhladze et al. reported a survival duration of 42 months in their ovarian cancer case (7). Roland and van Heerden reported that the longest survival within their cohort was 26 months (9).

Other studies either did not report survival outcomes or provided insufficient follow-up data.

Conducting meta-analysis was not feasible as a result of limited number of ovarian cancer cases, differences in study design and incomplete reporting of clinical outcomes. Therefore, the findings were summarised descriptively. Overall, the available literature suggests that pancreatic metastasis from ovarian cancer is exceptionally uncommon, with diagnosis often requiring careful radiological assessment and histological confirmation.

## Discussion

In the UK, ovarian cancer accounts for approximately 2% of all new cancer cases with around 7,700 new cases diagnosed per year, according to Cancer Research UK (https://www.cancerresearchuk.org/health-professional/cancer-statistics/statistics-by-cancer-type/ovarian-cancer#heading-Zero). Regardless of 5% decline in the European age-standardized incidence rates in the past decade (2006–2008 to 2016–2018), ovarian cancer remains a significant health burden. Surgical techniques and systemic anticancer (SACT) options have improved over time, yet advanced ovarian cancer continues to be associated with poor survival outcomes (12). It is responsible for approximately 4,100 deaths annually, averaging around 11 deaths per day from 2017 to 2019 as per the national cancer statistics (https://www.cancerresearchuk.org/health-professional/cancer-statistics/statistics-by-cancer-type/ovarian-cancer#heading-Zero^9^). Although the incidence has declined slightly, the disease’s high mortality underscores the need for improved treatment pathways, particularly in advanced stages (4).

Ovarian cancer primarily spreads within the peritoneal cavity through a process that differs significantly from hematogenous metastasis. Ovarian carcinoma cells often disseminate directly into the abdominal cavity, predominantly affecting the omentum, peritoneum and less frequently, distant organs such as the pancreas or the liver (4). The most common metastatic route is via direct extension to neighboring organs or through exfoliation of tumor cells, which are transported by peritoneal fluid to secondary sites; mainly intra-abdominal.

### Routes of metastasis and common sites

Ovarian cancer most commonly spreads within the peritoneal cavity. Tumor cells can detach from the ovarian surface, survive in peritoneal fluid as multicellular spheroids, and later implant on peritoneal and omental surfaces. For this reason, the omentum, diaphragm, bowel serosa, and pelvic peritoneum are among the most frequent sites of metastatic disease (4). Lymphatic spread is also recognized in epithelial ovarian cancer, including retroperitoneal nodal involvement (3).

Pancreatic involvement is rare, but it has been described in patients with ovarian malignancy, including isolated or complex pancreatic-region disease (7, 8, 10). In the present case, the exact route of spread cannot be confirmed. However, the pre-caval location of the lesion and its close relationship to the pancreatic head/uncinate region make lymphatic or locoregional spread plausible. A purely hematogenous route is less certain, as there was no clear evidence of widespread hematogenous visceral metastases when the pancreatic lesion was identified.

The pancreatic-region metastasis was clinically important because it caused hepatopancreatobiliary complications. The patient developed a tight common bile duct stricture requiring ERCP and pancreatic duct stent placement, and later developed cholangitis requiring antibiotics and supportive care. Similar reports show that pancreatic metastases may mimic a primary pancreatic tumor, making histological confirmation essential before deciding management (8, 10). This case also highlights the value of multidisciplinary care, including gynaecological oncology, hepatopancreatobiliary services, radiology, histopathology, medical oncology, endoscopy, and palliative care, to confirm the diagnosis, manage biliary complications, and guide treatment decisions.

#### Incidence of metastasis

Unfortunately, Ovarian cancer cases usually present at an advanced stage with metastasis within the peritoneal cavity. As described by Lengyel, approximately 75% of patients with ovarian cancer are diagnosed at an advanced stage when peritoneal spread is commonly already present (4). Stage IV ovarian cancer, characterized by distant metastases outside the peritoneal cavity to visceral organs such as to the liver, lungs, or pancreas, occurs in around 13% of newly diagnosed cases (4).

#### Treatment of stage IV ovarian cancer

Cytoreductive surgery, where all macroscopic tumor is removed, with ideally doublet peri-operative chemotherapy -neoadjuvant and adjuvant - regimen is the conventional treatment plan for patients with advanced ovarian cancer. During the last decade, maintenance therapies for platinum sensitive disease including bevacizumab and PolyADP-ribose polymerase (PARP) inhibitors have transformed the management of advanced ovarian cancer and demonstrated reduction in the recurrence/relapse rate hence prolonged progression-free survival (13). For the above patient and similar cases with pancreatic metastasis, it may be encouraging to offer further chemotherapy especially in the context of rapid progression and imminent biliary obstruction. Nevertheless, the degree of benefit is uncertain and often restricted by poor performance status, limited physiological reserve and patient wishes.

#### Outcome of stage IV disease

The outcome for patients with stage IV ovarian cancer remains bleak. The overall survival is significantly low due to many poor prognostic factors associated with such aggressive cancers; rapid proliferation, advanced stage at first presentation and its relative low chemosensitivity (14). Recurrence rate is significant and typically subsequent disease course is aggressive in spite of intensive treatment approach with cytoreductive surgery and chemotherapy (12). Poor prognostic outcome is associated with metastasis to vital organs including the pancreas, frequently treatment direction would be shifted towards symptoms management (4, 7, 8).

The patient’s journey highlights the significance of a multidisciplinary approach, integrating surgical, oncological and palliative care services, especially in advanced stages of ovarian cancer with atypical metastatic patterns.

This review has several limitations. The number of reported cases was small and most included studies were case reports or small retrospective series. Clinical details, imaging findings, treatment approaches and survival outcomes were not consistently reported across all studies. In addition, only English-language full-text articles were included, which may have introduced selection bias. These limitations prevented a meta-analysis and mean that the findings should be interpreted with caution.

## Conclusions

Metastatic involvement of the pancreas is an uncommon and unique clinical scenario that requires meticulous evaluation, particularly in patients who have previously been diagnosed with ovarian malignancy and are presenting with pancreatic lesion. This case emphasizes the importance of accurate clinical, radiological and histological assessment to distinguish metastatic disease from a second primary pancreatic tumor. Given the limited number of reported cases, there is no standard treatment approach and the management plan should be tailored to the individual patient with guidance from multidisciplinary team. Further reporting of similar cases is needed to improve understanding of this unusual pattern of spread and to guide future treatment decisions.

## Data Availability

The data supporting the conclusions of this article are included within the article. Further inquiries can be directed to the corresponding author. Raw clinical data are not publicly available due to patient confidentiality and privacy considerations.
